# Towards consensus on managing high mammographic density in population breast screening?

**DOI:** 10.1016/j.breast.2023.04.004

**Published:** 2023-05-03

**Authors:** Alberto Stefano Tagliafico, Nehmat Houssami

**Affiliations:** Radiology Section, Department of Health Sciences (DISSAL), University of Genova, Via Pastore 1, 16132, Genoa, Italy; Department of Radiology, IRCCS-Ospedale Policlinico San Martino, Largo Rosanna Benzi 10, 16132, Genoa, Italy; The Daffodil Centre, The University of Sydney, a Joint Venture with Cancer Council NSW, Sydney, New South Wales, Australia; Sydney School of Public Health, Faculty of Medicine and Health, The University of Sydney, Camperdown, New South Wales, Australia

In Medicine and in Radiology, impact on societal and health outcomes of a diagnostic pathway or methodology should be comprehensively investigated before implementing a screening approach for disease [1]. John R. Thornbury continued to develop this idea in his framework, the so-called “outcomes research” that includes a multidisciplinary approach for medical imaging usage, after Fryback and Thornbury proposed the critical assessment of the contribution of imaging to patient management though a hierarchical model [[2], [3], [4]]. At the highest level of the hierarchy of studies on diagnostic tests, according to a modified Thornbury scale societal impact, entailing a benefit–cost and cost-effectiveness analysis from a societal perspective, is mammography. Indeed, mammography is one of the few radiological tests directly shown to reduce death from breast cancer in randomized controlled trials [[5], [6], [7]].

The UK's Independent Panel on Breast Cancer Screening estimated from 11 randomized controlled trials, that mammographic screening conferred a 20% reduction in breast cancer mortality: the relative risk of breast cancer death for women invited (vs not invited) to screening was estimated as 0·80 (95% CI 0·73–0·89) [8]. For other breast imaging techniques, including MRI, mortality reduction has not been *directly* demonstrated to date. However, using data from the only RCT of MRI screening in dense breasts [9], a microsimulation analysis suggests that biennial MRI with mammography would further save lives amongst screened women [10,11]. Whether such an incremental health benefit, in terms of further mortality reduction from MRI as projected by modelling eventuates is yet unknown. Nonetheless, these data are relevant because mammography performs sub-optimally in women with increased fibroglandular tissue, specifically those with heterogeneously dense and extremely dense tissue (‘dense’ breasts).

In women with dense breasts, mammography sensitivity is reduced relative to non-dense breasts [[11], [12], [13]], with estimated lower screening benefit from a case–control study where screening performance was reported to be better in the fatty breasts group compared with the dense breasts group (overall sensitivity 75.7% vs 57.8%) [12]. Hence, the mortality reduction in this case-control study was estimated to be relatively smaller for women with dense breasts, with an odds ratio (OR) of 0.87 (95% CI 0.52–1.45) in the dense group and 0.59 (95% CI 0.44–0.79) in those with non-dense breasts.

In addition to being an independent risk factor for breast cancer, dense breast tissue appears radiopaque (white) on x-ray-based techniques, masking tumoral tissue [11,12,14,15]. Therefore, there is a higher risk of interval cancers in screened women who have dense breasts compared to women with non-dense breasts. Hence the concept of tailoring population screening for women with dense breasts to align with risk of breast cancer, whilst not entirely new, the broadening of this concept for density-only risk is gaining momentum. For high-risk women, such as those with >20% lifetime risk of breast cancer risk, annual breast MRI in addition to mammography has been recommended for some time [16]. Recently, the European Society of Breast Imaging (EUSOBI) recommended MRI screening for women with extremely dense breasts every two to four years [11] accounting for around 10% of women screened according to the density classification using the BI-RADS 5th edition. From a population perspective, this 10% represents a very high number of women, for whom MRI is unlikely to be feasible with current resources in most healthcare systems; importantly the recommendation does not consider other risk factors for breast cancer [17]. At present, the numbers of breast MRI that would be required if these recommendations are adopted may be far above what could actually be executed in MRI imaging settings even in highly developed countries, notwithstanding additional procedures and imaging follow-up that would be generated from density-driven MRI screening. Further, in the DENSE trial, which is a multicenter RCT performed in the Netherlands, 40 373 women (aged 50–75 years) with extremely dense breasts (as measured with automated software), were invited to undergo supplemental MRI(9): from 8061 participants invited to have breast MRI, 3279 (approximately 40%) did not undergo MRI (1872 declined to participate, others declined after the first round of screening, or were ineligible for MRI or did not respond). So, acceptability of MRI in population screening is moderate even in the context of an organized screening program. The DENSE RCT showed that MRI detected 16.5 per 1000 screenings (95% CI, 13.3 to 20.5) with a high recall rate of 94.9 per 1000 screenings – the latter raises genuine concern for potential implementation of supplemental MRI screening at a population level [9]. Therefore, alternative personalized approaches for screening women with dense breasts should be considered and formally evaluated, using an approach with more scalability and acceptability than MRI.

Potentially feasible options are to offer supplemental ultrasound, or tomosynthesis (in place of standard mammography) to women with dense breasts, or to reduce screening intervals, or to modify criteria for selecting those with extremely dense breasts (for example by combining density with additional risk factors for interval cancer) to streamline the number of women guided towards supplemental MRI screening [[17], [18]].

In this issue of the journal, Larsen and colleagues [15] offer new insights by retrospectively examining the potential impact on the interval cancer rate of annual versus biennial mammography screening in 508 536 screening exams. Amongst the study cohort of 213 105 women, 3125 cancers were screen-detected and 945 experienced an interval cancer. The goals of the study were to analyze time to interval cancer and breast density calculated automatically as a continuous measure (in percentage) to find cut-off values for supplemental screening or shorter screening intervals. The authors report that for women with volumetric breast density (>15.5%) the rate of interval cancer was high (4.1 per 1000), whereas in women with low volumetric breast density (<4.5%) the interval cancer rates were lower (0.4–3.0 per 1000 for Volumetric Glandular Density 1–3). These data could support the use of more intensive mammography screening for the subgroup of women with high breast density. Larsen and colleagues [15] suggest that annual mammography screening in women with high volumetric breast density could reduce interval cancer rate and would be a potential option where MRI is either unavailable, or of limited acceptability in the context of population screening. Ultrasound could be another option, with reported incremental cancer detection rates approximately 2–3 per 1000 but could be up to 4.90/1000 screens (95% CI: 3.21–7.19) when performed under optimal conditions. Incremental CDRs for supplemental MRI have been reported in the range of 14.2–16.5/1000 [19], much higher than US, but these are associated with high recall and biopsy rates (9.5%–15.9%, up to 69/1000 screens) [20,21]. Relative to the quantified cancer detection rates both US and MRI appear to have a modest effect in reducing ICR [19] but MRI has been shown to significantly reduce interval cancer rate in the RCT [9].

In the era of personalized medicine, tailoring screening and diagnostic approaches according to the risk profile of individuals would be ideal, but the road (and required evidence base) to adopting this concept for population screening is far from clear. On the basis of evidence from the Dense RCT, the recommendation for broader offer of MRI screening for those with dense breasts may be considered premature by some but tardy by others – what cannot be contested however is that screening tests should be acceptable, accessible and non-invasive, and not merely sensitive. Screening programs are ideally positioned to evaluate effective and scalable measures to address the limitations of screening mammography in those at most risk of experiencing an interval cancer, including but not limited to those with dense breasts [17]. Introducing annual mammography screening instead of biennial screening for women with very high breast density could reduce the risk of interval cancer as stated by Larsen and colleagues [15] and may be more feasible than MRI, but other options as already articulated could be explored and evaluated. Essential to any proposed change to breast screening is careful examination of how this would impact the trade-off between benefits and harms and ensuring that evolution of screening for breast cancer does not widen inequity in access to healthcare. We are far from consensus on personalized breast cancer screening for high breast density, RCT and real-world population data can guide new screening strategies, as does the voice of the community. Scalability, however, should not be understated in the pathway towards tailored screening for high breast density or any other risk-guided care.
